# Innate immune changes s in aging and in older T2DM subjects with or without obesity

**DOI:** 10.1093/geroni/igaf122.2880

**Published:** 2025-12-31

**Authors:** Tamas Fulop, Abdelouahed Khalil, Jacek Witkowski, José Morais

**Affiliations:** Université de Sherbrooke, Sherbrooke, Quebec, Canada; Université de Sherbrooke, Sherbrooke, Quebec, Canada; University Medical School of Gdansk, Gdansk, Pomorskie, Poland; McGill University, Montréal, Quebec, Canada

## Abstract

It could be that in older adults with diabetes mellitus type 2 (T2DM), especially associated with obesity (“diabesity”), their innate immune system changes would be more accentuated than in normal aging. To investigate the monocytes and macrophages phenotypes and functions.

**Methods:**

We recruited 48 older subjects (aged from 65 to 80 years), 16 as healthy controls, 16 non obese and 16 obese T2DM patients. Obesity was determined by BMI over 30. We separated the monocytes by adherence and studied their phenotypes by using CD14 and CD16 and diferentiated them in macropages. The monocytes phenotype distribution in older and T2DM non obese subjects resembled that in young subjects, while T2DM obese subjects had a significant increase in the intermediate subpopulation (pro-inflammatory). When monocytes were incubated with M-CSF or autologous sera, they became predominantly M2 in healthy and T2DM non-obese subjects, while GM-CSF induced M1 phenotype in healthy and non-obese T2DM subjects. The M2 had an increased phagocytic capacity but produced less ROS. In contrast the M1 had a great ROS production capacity but much less phagocytic activity. Macrophages of obese T2DM subjects incubated in autologous sera differentiated in M1 in contrast to the healthy and non-obese T2DM subjects, and produced a significantly higher amount of ROS after opsonised E.coli and LPS stimulation. TNFα is increased in both T2DM group compared to healthy subjects. There is an inflammatory status with aging which is not modified by T2DM but much increased when obesity is present concomitantly with T2DM.

